# Supplementary material for: Co-production of an educational package for the universal human papillomavirus (HPV) vaccination programme tailored for schools with low uptake: a participatory study protocol
Source: BMJ Open. 2020 Nov 4;10(11):e039029. doi: 10.1136/bmjopen-2020-039029 (PMC7643513; doi:10.1136/bmjopen-2020-039029)
Supplement: Author's manuscript [file bmjopen-2020-039029.draft_revisions.pdf]

BMJ Open is committed to open peer review. As part of this commitment we make the peer review history of every article we publish publicly available.

When an article is published we post the peer reviewers' comments and the authors' responses online. We also post the versions of the paper that were used during peer review. These are the versions that the peer review comments apply to.

The versions of the paper that follow are the versions that were submitted during the peer review process. They are not the versions of record or the final published versions. They should not be cited or distributed as the published version of this manuscript.

BMJ Open is an open access journal and the full, final, typeset and author-corrected version of record of the manuscript is available on our site with no access controls, subscription charges or pay-per-view fees (http://bmjopen.bmj.com).

If you have any questions on BMJ Open's open peer review process please email info.bmjopen@bmj.com

BMJ Open

Co-production of an educational package for the universal human papillomavirus (HPV) vaccination programme tailored for schools with low uptake: A participatory study protocol

Journal:	*BMJ Open*
Manuscript ID	bmjopen-2020-039029
Article Type:	Protocol
Date Submitted by the Author:	02-Apr-2020
Complete List of Authors:	Fisher, Harriet; University of Bristol Faculty of Health Sciences, Bristol Medical School<br>Audrey, Suzanne; University of Bristol, Population Health Sciences<br>Chantler, Tracey; London School of Hygiene & Tropical Medicine, Global Health & Development<br>Finn, Adam; University of Bristol, Division of Clinical Sciences South Bristol<br>Letley, Louise; Public Health England, National Infection Service<br>Mounier-Jack, Sandra; London School of Hygiene and Tropical Medicine,<br>Thomas, Clare; University of Bristol, Centre for Academic Primary Care<br>Yates, Julie; Public Health England<br>Hickman, Matthew; University of Bristol,
Keywords:	PUBLIC HEALTH, EDUCATION & TRAINING (see Medical Education & Training), Paediatric infectious disease & immunisation < PAEDIATRICS

SCHOLARONE™
Manuscripts

I, the Submitting Author has the right to grant and does grant on behalf of all authors of the Work (as defined in the below author licence), an exclusive licence and/or a non-exclusive licence for contributions from authors who are: i) UK Crown employees; ii) where BMJ has agreed a CC-BY licence shall apply, and/or iii) in accordance with the terms applicable for US Federal Government officers or employees acting as part of their official duties; on a worldwide, perpetual, irrevocable, royalty-free basis to BMJ Publishing Group Ltd ("BMJ") its licensees and where the relevant Journal is co-owned by BMJ to the co-owners of the Journal, to publish the Work in this journal and any other BMJ products and to exploit all rights, as set out in our licence.

The Submitting Author accepts and understands that any supply made under these terms is made by BMJ to the Submitting Author unless you are acting as an employee on behalf of your employer or a postgraduate student of an affiliated institution which is paying any applicable article publishing charge ("APC") for Open Access articles. Where the Submitting Author wishes to make the Work available on an Open Access basis (and intends to pay the relevant APC), the terms of reuse of such Open Access shall be governed by a Creative Commons licence – details of these licences and which Creative Commons licence will apply to this Work are set out in our licence referred to above.

Other than as permitted in any relevant BMJ Author's Self Archiving Policies, I confirm this Work has not been accepted for publication elsewhere, is not being considered for publication elsewhere and does not duplicate material already published. I confirm all authors consent to publication of this Work and authorise the granting of this licence.

For peer review only

TITLE PAGE

Co-production of an educational package for the universal human papillomavirus (HPV) vaccination programme tailored for schools with low uptake: A participatory study protocol

Harriet Fisher, Population Health Sciences, Bristol Medical School, University of Bristol

Suzanne Audrey, Population Health Sciences, Bristol Medical School, University of Bristol

Tracey Chantler, Faculty of Public Health and Policy, London School of Hygiene and Tropical Medicine

Adam Finn, School of Cellular and Molecular Medicine, University of Bristol
of Bristol

Louise Letley, National Infection Service, Public Health England

Sandra Mournier-jack, Faculty of Public Health and Policy, London School of Hygiene and Tropical Medicine

Clare Thomas, Population Health Sciences, Bristol Medical School, University

Julie Yates, Lead Consultant for Screening and Immunisation, South West, Public Health England

Matthew Hickman, Population Health Sciences, Bristol Medical School, University of Bristol

Corresponding author:

Dr Harriet Fisher

Senior Research Associate

Population Health Sciences, Bristol Medical School

39 Whatley Road

Bristol BS8 2PS

Email: Harriet.Fisher@bristol.ac.uk

Telephone: +44 (0) 117 928 7223

Main text word count: 2,043

Key words: HPV vaccine; participatory study; co-production; young people; educational

For peer review only - http://bmjopen.bmj.com/site/about/guidelines.xhtml

ABSTRACT

Aim

To co-produce with young people an educational package about the human papillomavirus (HPV) vaccine that is tailored to increase vaccine uptake in schools and populations with lower uptake.

Introduction

Persistent infection with HPV can result in cancers affecting men and especially women. From September 2019, the English schools-based HPV vaccination programme will be expanded to include young men (in addition to young women) aged 12 to 13 years. Some young people attending schools with lower uptake of the vaccine have unmet information needs. We hypothesise that mechanisms to address information needs and increase young people's autonomy in consent procedures will result in higher uptake.

Methods and Analysis

The Medical Research Council's framework for development and evaluation of complex interventions will inform intervention development. Recruitment of young people aged 12 to 15 years and key stakeholders (National Health Service (NHS) commissioners, school staff, immunisation nurses, youth workers/practitioners) will be facilitated through existing links with healthcare organisations, schools and youth organisations in areas with lower uptake of the HPV vaccination programme. The proposed research will comprise three phases: (i) a rapid review of adolescent immunisation materials and preliminary qualitative interviews with young people and key stakeholders; (ii) theory development and co-production of HPV vaccine communication materials through an iterative process with young people, and; (iii) testing delivery mechanisms and acceptability of the educational package in four schools with lower uptake.

Ethics and Dissemination

The University of Bristol's Faculty of Health Sciences Research Ethics Committee provided approvals for the study. A dissemination event for young people and key stakeholders and webinar with the National Immunisation Network will be organised. The study findings will be published in peer-reviewed journals and presented at conferences. Recommendations for a future larger-scale study will be made.

Word count: 295

For peer review only

For peer review only - http://bmjopen.bmj.com/site/about/guidelines.xhtml

ARTICLE SUMMARY

We have previously identified that some young people have unmet information needs about the HPV vaccination programme. There is currently a lack of evidence for the effectiveness of educational interventions about the HPV vaccination programme targeted at schools with lower uptake. This research protocol proposes an innovative, participatory research approach to develop with young people an educational package that aims to increase uptake by addressing young people's information needs and autonomy in decision-making. The study team represent a collaboration between academic researchers and public health practitioners who will work together to produce recommendations for a future intervention study testing effectiveness. Although this research focuses on the HPV vaccination programme, the findings may be relevant to other vaccines that are offered to adolescents in school settings.

STRENGTHS AND LIMITATIONS OF THIS STUDY

- This study aims to co-produce with young people an educational package that addresses young people's information needs about the HPV vaccination programme.
- The protocol for this study documents the use of an innovative, participatory methodological approach.
- Communication materials will be iteratively designed by the co-production team taking into account young people's views at all stages of the development cycle.
- Findings from this study will be used to make recommendations for a future larger-scale study.
- Parents, who also have unmet information needs, are not included in this study.

For peer review only - http://bmjopen.bmj.com/site/about/guidelines.xhtml

INTRODUCTION

The human papillomavirus (HPV) vaccine currently used in England protects against infection from high-risk HPV types 16 and 18 which cause cancers affecting the cervix, vulva, vagina, penis, anus, and oral cavity. The vaccine also protects against types 6 and 11 which cause 90% of genital warts. High coverage of the English HPV vaccination programme for young women aged 12 to 13 years has been achieved. Recent evidence for population-level effectiveness has highlighted the potential for HPV vaccination programmes to eradicate cervical cancer [1, 2].

However, national data often conceal within-country inequalities in uptake and access. Without concerted efforts to address lower uptake amongst some populations, existing disparities in the incidence of cervical cancer by ethnicity and socioeconomic deprivation may increase [3-5]. Wide variations in uptake of the HPV vaccination programme across local authorities exist (range: 70.2-95.8% for the first dose in 2018/19) [6]. We previously identified lower uptake by area and amongst some population groups, including minority ethnic groups [7]. Our research in schools with lower uptake showed complex socio-cultural factors influence beliefs and priorities for young women to be vaccinated [8]. The requirement for written parental consent also acts as a major barrier [9], which can be overcome to some extent by enabling verbal parental consent and adolescent self-consent [10][Fisher et al, Extent of young women's autonomy in the HPV vaccination programme and influence of information communication: A qualitative study].

Based on emerging evidence, the Joint Committee on Vaccination and Immunisation concluded that extending the HPV vaccination programme to include young men could be cost-effective [11]. The Department of Health announced the inclusion of young men from the 2019/20 programme year. If sufficient uptake is attained, a universal vaccination programme will strengthen England's position to eliminate some HPV types and HPV-related cancers in the longer term. Men who have sex with men will also receive optimal protection by immunisation ahead of sexual debut [11, 12].

It is currently unknown whether the universal HPV vaccination programme will be delivered equitably by gender, or whether existing inequalities in uptake and incidence of HPV-related disease among some

For peer review only - http://bmjopen.bmj.com/site/about/guidelines.xhtml

groups will be decreased or widened [13]. Inequalities may prevent delivery of the programme as intended, with insufficient understanding and engagement of young people and their parents or carers. Despite largely positive public attitudes to vaccination [14], recent calls have been made to tackle negative misconceptions of vaccines and limit health misinformation circulating in social media [15]. Many young people have limited or no understanding of the vaccines they receive or the diseases they are intended to prevent [16]. Our previous research confirms some young women attending schools with lower uptake have unmet information needs, which can have implications for obtaining consent and vaccination uptake [8][Fisher et al, Extent of young women's autonomy in the HPV vaccination programme and influence of information communication: A qualitative study]. A systematic review, including two UK-based studies, showed young men have lower knowledge and understanding of HPV than their female counterparts [17].

Methods to increase understanding, suggested by families and professionals, include: healthcare professional advocacy, accessible information formats, and delivery of educational sessions, in addition to the routine provision of information leaflets [Fisher et al, Extent of young women's autonomy in the HPV vaccination programme and influence of information communication: A qualitative study]. Preliminary feedback from young people suggests the delivery of interactive HPV vaccine awareness raising sessions by a researcher in the school-setting are acceptable. Young people's questions during the sessions related both to the HPV vaccine and preparation for the vaccination session.

Internationally, educational interventions about the HPV vaccine have been developed through research. These have targeted young adults [18, 19], parents [20-22] and professionals [20-22]. A complex intervention delivered in a United States of America healthcare setting, comprising an educational component delivered to young people aged 11 to 21 years, was shown to be effective at increasing uptake [23]. Similarly, a schools-based educational programme delivered to Swedish students aged 16 years was shown to increase self-reported uptake of the HPV vaccine [24]. In Australia, a complex schools-based intervention was not shown to be effective at increasing vaccination uptake, but improved adolescent knowledge and psychosocial outcomes [25].

For peer review only

For peer review only - http://bmjopen.bmj.com/site/about/guidelines.xhtml

These studies suggest that an educational-based intervention to increase uptake has potential to be an acceptable and effective intervention in the context of the English universal HPV vaccination programme. We hypothesise that mechanisms to address information needs and increase young people's autonomy in consent procedures will result in higher uptake of the programme. We are unaware of any other educational packages that have been developed specifically for areas and populations groups with lower uptake [7], or that have tested these specific mechanisms to increase uptake.

The aim of the study is to co-produce jointly with young people an HPV vaccination educational package that can be used within the adolescent schools-based immunisation programme and is tailored at increasing vaccine uptake in areas and populations with lower HPV vaccination coverage.

METHODS AND ANALYSIS

We propose an innovative, participatory research approach involving co-production of an education package about the HPV vaccine with young people. The 2008 Medical Research Council (MRC) guidance for complex intervention development and evaluation will be followed [26].

Patient and public involvement

The design of this study was informed by our qualitative research undertaken with young women, parents, immunisation nurses and school staff [Fisher et al, Extent of young women's autonomy in the HPV vaccination programme and influence of information communication: A qualitative study]. We have consulted with the Bristol Young People's Advisory Group (YPAG) and Bristol City Youth Council about the design of the study. The Bristol YPAG comprises young people aged ten to 17 years who are interested in healthcare and research. The Bristol City Youth Council are an elected group of 28 young people from across the city. Both groups have been consulted about the design of the study and participant materials. They will also be invited to an event at the end of the study to consider findings and recommendations with the young people, healthcare professionals and school staff involved in the study.

For peer review only - http://bmjopen.bmj.com/site/about/guidelines.xhtml

During Stages One and Two, young men and women aged 12 to 15 years will be recruited from two youth community organisations in the south west of England. These organisations work with young people with similar sociodemographic backgrounds to the participants of our previous studies undertaken in schools with lower uptake. An additional youth community organisation in London will also be recruited. The age group 12 to 15 years is selected as these young people will have diverse experiences in relation to the HPV vaccination programme.

The study will comprise three phases: (1) a rapid review of adolescent immunisation information materials and preliminary qualitative interviews with young people and key stakeholders; (2) theory development and co-production of HPV vaccine communication materials in an iterative process with young people, and; (3) examining delivery mechanisms and acceptability of the educational package in four schools with lower uptake of the HPV vaccination programme.

Stage One

A scoping review of existing young people's immunisation information materials will be undertaken to inform the content of the initial workshop. Our qualitative research findings suggest that young people value external providers delivering health-related information in school settings. Preliminary individual or paired/peer group interviews (depending on the preference of participants) with young people (n~6) and key stakeholders (commissioners, school staff, immunisation nurses and youth workers/practitioners) (n~6) will be undertaken to explore further who should deliver the educational package and which key messages to cover.

Stage Two

Drawing on our previous qualitative research findings, it is envisaged that the educational package will include question and answer sessions alongside a series of short videos. Information needs may differ between schools depending on the sociodemographic characteristics of the student population. Therefore, additional videos will be co-produced to allow the educational session to be tailored as appropriate (e.g. provision of information in multiple languages, additional information for parents on vaccine safety).

For peer review only - http://bmjopen.bmj.com/site/about/guidelines.xhtml

For peer review only

The materials will be co-produced by young people (n~6) and creative designers who are experienced in working with young people to create health-related media materials.

An initial workshop with young people (n~6-8) will be organised at a community youth organisation where they will be asked to: (i) review existing communication materials; (ii) comment on their understanding of key HPV vaccine messages; (iii) make suggestions of their preferred messages, design and language style, and; (iv) propose the type of media to be used (e.g. animation, vloggers). Platforms to distribute the communication materials (e.g. videos, power-points) which are accessible by young people will be explored. Advice will also be sought from voluntary organisations, immunisation teams and school staff.

At least three additional workshops will be organised in different community youth organisations. Here the materials will be iteratively redesigned by the co-production team. Young people's views will be taken into account at all stages of the development cycle.

Stage Three

Four schools with lower uptake of the HPV vaccination programme will be recruited to the study. It is envisaged that the educational package will last approximately one hour and be delivered to groups of vaccine eligible young people (aged 12-13 years). The package will be piloted approximately two weeks ahead of the scheduled vaccination session. Methods for obtaining young people's feedback will be explored (e.g. short paper-based questionnaires, interactive quiz). Following the question and answer session, young people will be provided with consent forms and promotional materials available in different languages. Young people will be encouraged to share these with their parents/carers and return the completed consent form.

The feasibility of obtaining information from immunisation teams relating to HPV vaccine uptake by gender, ethnicity, deprivation, and types of consent routinely obtained (no response, parent refusal, written or verbal parental consent, young person refusal or self-consent) will be assessed for a future study.

For peer review only

For peer review only - http://bmjopen.bmj.com/site/about/guidelines.xhtml

A purposive sample of young people from participating schools (~12) and key stakeholders (n~12) will be invited to take part in semi-structured or paired/peer group interviews (depending on the preference of participants) to gather information related to intervention acceptability, understanding of the HPV vaccine, and perceptions of adolescent autonomy during consent. The feasibility and sustainability of who delivers the education package will be further explored.

Analysis

All interview recordings will be transcribed verbatim and any potentially identifying information removed. Familiarisation with the data will involve two researchers reading and discussing the transcripts to compare and begin to code and categorise the data. Thematic analysis [27] will be undertaken, within which similarities and differences will be explored. Data analysis will be assisted by the QSR NVivo12 software package.

Together, the findings from the rapid review and qualitative research will inform the logic model and development of theory for the educational package.

The outcome from this participatory research study will be a tailored educational intervention about the universal HPV vaccination programme. We will seek additional funding to test whether the educational package can: (i) improve uptake of the vaccination programme in schools/areas with lower uptake; (ii) reduce inequalities in programme delivery; (iii) increase young people's understanding about the HPV vaccine, and; (iv) increase young people's autonomy in decision-making and consent procedures.

Ethics and dissemination

The University of Bristol's Faculty of Health Sciences Research Ethics Committee have provided ethical approvals for the study (reference: 99102).

We will disseminate this research to the academic community, health and youth work practitioners and health policy workers. For the academic community we plan to submit at least one peer-reviewed paper. We will also disseminate the results to at least one national and one international conference. For

For peer review only - http://bmjopen.bmj.com/site/about/guidelines.xhtml

practitioners we will complete a report of our findings and will disseminate the results through practitioner conferences and events.

We will also hold a community stakeholder event to which we will invite school nurses, school staff, parents and young people. This will be an opportunity to discuss the educational package for the HPV vaccine. Healthcare professionals will be actively encouraged to be involved in the dissemination activities, and findings from the study will also be disseminated through appropriate websites and social media. The final report of the study will also provide an opportunity for further dissemination.

For peer review only

REFERENCES

1. Drolet, M., et al., Population-level impact and herd effects following the introduction of human papillomavirus vaccination programmes: updated systematic review and meta-analysis. The Lancet, 2019. **394**(10197): p. 497-509.

2. Simms, K., et al., Impact of scaled up human papillomavirus vaccination and cervical screening and the potential for global elimination of cervical cancer in 181 countries, 2020–99: a modelling study. The Lancet Oncology, 2019. **20**(3): p. 394-407.

3. Johnson, H., et al., Effect of HPV vaccination and cervical cancer screening in England by ethnicity: a modelling study. The Lancet Public Health, 2018. **3**(1): p. e44-e51.

4. Baker, D. and E. Middleton, Cervical screening and health inequality in England in the 1990s. 2003. **57**(6): p. 417-423.

5. Shack, L., et al., Variation in incidence of breast, lung and cervical cancer and malignant melanoma of skin by socioeconomic group in England. BMC Cancer, 2008. **8**(1): p. 271.

6. Public.Health.England, HPV vaccination coverage in adolescent females in England: 2018 to 2019, 2019. Available from: https://www.gov.uk/government/statistics/hpv-vaccination-coverage-in-adolescent-females-in-england-2018-to-2019.

7. Fisher, H., et al., Examining inequalities in the uptake of the school-based HPV vaccination programme in England: a retrospective cohort study. Journal of Public Health, 2013. **36**(1): p. 36-45.

8. Batista Ferrer, H., et al., Barriers and facilitators to uptake of the school-based HPV vaccination programme in an ethnically diverse group of young women. Journal of Public Health, 2016. **38**(3): p. 569-577.

9. Batista Ferrer, H., Factors influencing the uptake of the Human Papillomavirus (HPV) vaccination programme. Thesis (Ph.D.), 2014.

10. Audrey, S., et al., Impact and acceptability of self-consent procedures for the school-based human papillomavirus vaccine: a mixed-methods study protocol. BMJ Open, 2018. **8**(3): p. e021321.

11. Joint.Committee.on.Vaccination.and.Immunisation, 2018. Available from: https://www.gov.uk/government/publications/jcvi-statement-extending-the-hpv-vaccination-programme-conclusions.

For peer review only - http://bmjopen.bmj.com/site/about/guidelines.xhtml

12. Kesten, J., Flannagan, C, Ruane-McAteer, E, Merriel, S, Nadarzynski, T, Shapiro, G, Rosberger, Z & Prue, G, A mixed-methods study in England and Northern Ireland to understand young men-who-have-sex-with-men's knowledge and attitude towards human papillomavirus vaccination. BMJ Open, 2019. **9**: p. e025070.

13. Department of Health & Social Care, Equality Analysis: Human Papillomavirus (HPV) Vaccination, 2018. Available from: https://assets.publishing.service.gov.uk/government/uploads/system/uploads/attachment_data/file/758777/HPV_Boys_Equality_Assessment.pdf.

14. Campbell, H., et al., Changing attitudes to childhood immunisation in English parents. Vaccine, 2017. **35**(22): p. 2979-2985.

15. Royal Society for Public Health, Moving the needle: promoting vaccination uptake across the life course, 2018. Available from: https://www.rsph.org.uk/uploads/assets/uploaded/f8cf580a-57b5-41f4-8e21de333af20f32.pdf.

16. Davies, C., et al., 'Is it like one of those infectious kind of things?' The importance of educating young people about HPV and HPV vaccination at school. Sex Education, 2017. **17**(3): p. 256-275.

17. Prue, G., et al., Knowledge and acceptance of human papillomavirus (HPV) and HPV vaccination in adolescent boys worldwide: A systematic review. Journal of Cancer Policy, 2016. **10**: p. 1-15.

18. Ayoub, N., J. Sunwoo, and H. Starmer, Implementation of a targeted HPV educational program in a population with HIV. World Journal of Otorhinolaryngology - Head and Neck Surgery, 2019.

19. Chang, I., et al., Effect of an educational intervention on HPV knowledge and vaccine attitudes among urban employed women and female undergraduate students in China: a cross-sectional study. BMC Public Health, 2013. **13**(1): p. 916.

20. Reiter, P., et al., HPV and HPV vaccine education intervention: effects on parents, healthcare staff, and school staff. Cancer epidemiology, biomarkers & prevention, 2011. **20**(11): p. 2354-2361.

21. Dempsey, A., et al., Effect of a Health Care Professional Communication Training Intervention on Adolescent Human Papillomavirus Vaccination: A Cluster Randomized Clinical Trial. JAMA Pediatrics, 2018. **172**(5): p. e180016-e180016.

22. Paskett, E., et al., Results of a Multilevel Intervention Trial to Increase Human Papillomavirus (HPV) Vaccine Uptake among Adolescent Girls. Cancer Epidemiology Biomarkers & Prevention, 2016. **25**(4): p. 593-602.

23. Perkins, R., et al., Effectiveness of a provider-focused intervention to improve HPV vaccination rates in boys and girls. Vaccine, 2015. **33**(9): p. 1223-9.

24. Grandahl, M., et al., School-based intervention for the prevention of HPV among adolescents: a cluster randomised controlled study. BMJ Open, 2016. **6**(1): p. e009875.

25. Skinner, S., et al., LB1.1 Randomised controlled trial of a complex intervention to improve school-based hpv vaccination for adolescents: the hpv. edu study. Sexually Transmitted Infections, 2015. **91**(Suppl 2): p. A77-A77.

26. Craig, P., et al., Developing and evaluating complex interventions: the new Medical Research Council guidance. BMJ (Clinical research ed.), 2008. **337**: p. a1655-a1655.

For peer review only

27. Braun, V. and V. Clarke, Using thematic analysis in psychology. Qualitative Research in Psychology, 2006. **3**(2): p. 77-101.

For peer review only

For peer review only - http://bmjopen.bmj.com/site/about/guidelines.xhtml

Data statement

It is the authors' intention to share their underpinning research data in order to maximise reuse and evidence their findings. When participants consent or assent to take part in the study, their permission to deposit anonymised transcripts at the University of Bristol Research Data Repository (data.bris.ac.uk/data) will be sought. A metadata record will be published openly by the repository and this record will clearly state how data can be accessed by bona fide researchers. Requests for access will be directed to the Research Data team at Bristol, who will assess the motives of potential data re-users before granting access to the data. No authentic request for access will be refused and re-users will not be charged for any part of this process.

Author statement

All authors were involved in the conception and design of the research: MH is Principal Investigator and contributes expertise in researching infectious disease control; SA contributes expertise in qualitative research and process evaluation. TC provides implementation, mixed methods and anthropology research expertise in relation to UK vaccination programmes. AF provides experience in the field of vaccine and policy development. LL provides expertise in implementing the national HPV vaccination programme. SM-J has expertise in health systems and policy evaluations, notably vaccination programmes. CT offers expertise in knowledge mobilisation. JY will support the dissemination of findings and wider implementation of the educational package as appropriate. As study manager, HF will be responsible for co-ordinating the study and provides links with community organisations and schools. HF wrote the first draft and all authors contributed to the final version of the manuscript.

Funding

This work is supported by the Medical Research Council (MRC) Public Health Intervention Development scheme (project number: MR/T027150/1).

For peer review only - http://bmjopen.bmj.com/site/about/guidelines.xhtml

Acknowledgments

The study is supported by the NIHR Health Protection Research Unit in Behavioural Science and Evaluation at the University of Bristol. The views expressed are those of the authors and not necessarily those of the NHS, the NIHR, the Department of Health or Public Health England.

The support of The Centre for the Development and Evaluation of Complex Interventions for Public Health Improvement (DECIPHer), a UKCRC Public Health Research Centre of Excellence. Joint funding (MR/KO232331/1) from the British Heart Foundation, Cancer Research UK, Economic and Social Research Council, Medical Research Council, the Welsh Government and the Wellcome Trust, under the auspices of the UK Clinical Research Collaboration, is gratefully acknowledged.

Conflicts of interest

The authors declare there are no conflicts of interest.

For peer review only

For peer review only - http://bmjopen.bmj.com/site/about/guidelines.xhtml

BMJ Open

Co-production of an educational package for the universal human papillomavirus (HPV) vaccination programme tailored for schools with low uptake: A participatory study protocol

Journal:	*BMJ Open*
Manuscript ID	bmjopen-2020-039029.R1
Article Type:	Protocol
Date Submitted by the Author:	01-Sep-2020
Complete List of Authors:	Fisher, Harriet; University of Bristol Faculty of Health Sciences, Bristol Medical School<br>Audrey, Suzanne; University of Bristol, Population Health Sciences<br>Chantler, Tracey; London School of Hygiene & Tropical Medicine, Global Health & Development<br>Finn, Adam; University of Bristol, Division of Clinical Sciences South Bristol<br>Letley, Louise; Public Health England, National Infection Service<br>Mounier-Jack, Sandra; London School of Hygiene and Tropical Medicine,<br>Thomas, Clare; University of Bristol, Centre for Academic Primary Care<br>Yates, Julie; Public Health England<br>Hickman, Matthew; University of Bristol,
**Primary Subject Heading**:	Public health
Secondary Subject Heading:	Qualitative research, Public health
Keywords:	PUBLIC HEALTH, EDUCATION & TRAINING (see Medical Education & Training), Paediatric infectious disease & immunisation < PAEDIATRICS

SCHOLARONE™
Manuscripts

I, the Submitting Author has the right to grant and does grant on behalf of all authors of the Work (as defined in the below author licence), an exclusive licence and/or a non-exclusive licence for contributions from authors who are: i) UK Crown employees; ii) where BMJ has agreed a CC-BY licence shall apply, and/or iii) in accordance with the terms applicable for US Federal Government officers or employees acting as part of their official duties; on a worldwide, perpetual, irrevocable, royalty-free basis to BMJ Publishing Group Ltd ("BMJ") its licensees and where the relevant Journal is co-owned by BMJ to the co-owners of the Journal, to publish the Work in this journal and any other BMJ products and to exploit all rights, as set out in our licence.

The Submitting Author accepts and understands that any supply made under these terms is made by BMJ to the Submitting Author unless you are acting as an employee on behalf of your employer or a postgraduate student of an affiliated institution which is paying any applicable article publishing charge ("APC") for Open Access articles. Where the Submitting Author wishes to make the Work available on an Open Access basis (and intends to pay the relevant APC), the terms of reuse of such Open Access shall be governed by a Creative Commons licence – details of these licences and which Creative Commons licence will apply to this Work are set out in our licence referred to above.

Other than as permitted in any relevant BMJ Author's Self Archiving Policies, I confirm this Work has not been accepted for publication elsewhere, is not being considered for publication elsewhere and does not duplicate material already published. I confirm all authors consent to publication of this Work and authorise the granting of this licence.

For peer review only

TITLE PAGE

Co-production of an educational package for the universal human papillomavirus (HPV) vaccination programme tailored for schools with low uptake: A participatory study protocol

Harriet Fisher, Population Health Sciences, Bristol Medical School, University of Bristol

Suzanne Audrey, Population Health Sciences, Bristol Medical School, University of Bristol

Tracey Chantler, Faculty of Public Health and Policy, London School of Hygiene and Tropical Medicine

Adam Finn, School of Cellular and Molecular Medicine, University of Bristol
of Bristol

Louise Letley, National Infection Service, Public Health England

Sandra Mounier-Jack, Faculty of Public Health and Policy, London School of Hygiene and Tropical Medicine

Clare Thomas, Population Health Sciences, Bristol Medical School, University

Julie Yates, Lead Consultant for Screening and Immunisation, South West, Public Health England

Matthew Hickman, Population Health Sciences, Bristol Medical School, University of Bristol

Corresponding author:

Dr Harriet Fisher

Senior Research Associate

Population Health Sciences, Bristol Medical School

39 Whatley Road

Bristol BS8 2PS

Email: Harriet.Fisher@bristol.ac.uk

Telephone: +44 (0) 117 928 7223

Main text word count: 2,190

Key words: HPV vaccine; participatory study; co-production; young people; educational

For peer review only

For peer review only - http://bmjopen.bmj.com/site/about/guidelines.xhtml

ABSTRACT

Aim

To co-produce with young people an educational package about the human papillomavirus (HPV) vaccine that is tailored to increase vaccine uptake in schools and populations with lower uptake.

Introduction

Persistent infection with HPV can result in cancers affecting men and especially women. From September 2019, the English schools-based HPV vaccination programme will be expanded to include young men (in addition to young women) aged 12 to 13 years. Some young people attending schools with lower uptake of the vaccine have unmet information needs. We hypothesise that mechanisms to address information needs and increase young people's autonomy in consent procedures will result in higher uptake.

Methods and Analysis

The Medical Research Council's framework for development and evaluation of complex interventions will inform intervention development. Recruitment of young people aged 12 to 15 years and key stakeholders (National Health Service (NHS) commissioners, school staff, immunisation nurses, youth workers/practitioners) will be facilitated through existing links with healthcare organisations, schools and youth organisations in areas with lower uptake of the HPV vaccination programme. The proposed research will comprise three phases: (i) a rapid review of adolescent immunisation materials and preliminary qualitative interviews with young people and key stakeholders; (ii) theory development and co-production of HPV vaccine communication materials through an iterative process with young people, and; (iii) testing delivery mechanisms and acceptability of the educational package in four schools with lower uptake.

Ethics and Dissemination

The University of Bristol's Faculty of Health Sciences Research Ethics Committee provided approvals for the study. A dissemination event for young people and key stakeholders and webinar with the National Immunisation Network will be organised. The study findings will be published in peer-reviewed journals and presented at conferences. Recommendations for a future larger-scale study will be made.

Word count: 289

For peer review only

ARTICLE SUMMARY

STRENGTHS AND LIMITATIONS

1. This study aims to co-produce with young people an educational package that addresses young people's information needs about the HPV vaccination programme.
2. The protocol for this study documents the use of an innovative, participatory methodological approach.
3. Communication materials will be iteratively designed by the co-production team, taking into account young people's views at all stages of the development cycle.
4. Findings from this study will be used to make recommendations for a future larger-scale study.
5. Parents, who also have unmet information needs, are not included in this study.

For peer review only

For peer review only - http://bmjopen.bmj.com/site/about/guidelines.xhtml

INTRODUCTION

The human papillomavirus (HPV) vaccine currently used in England protects against infection from high-risk HPV types 16 and 18 which cause cancers affecting the cervix, vulva, vagina, penis, anus, and oral cavity. The vaccine also protects against types 6 and 11 which cause 90% of genital warts. High coverage of the English HPV vaccination programme for young women aged 12 to 13 years has been achieved. Recent evidence for population-level effectiveness has highlighted the potential for HPV vaccination programmes to eradicate cervical cancer [1, 2].

However, national data often conceal within-country inequalities in uptake and access. Without concerted efforts to address lower uptake amongst some populations, existing disparities in the incidence of cervical cancer by ethnicity and socioeconomic deprivation may increase [3-5]. Wide variations in uptake of the HPV vaccination programme across local authorities exist (range: 70.2-95.8% for the first dose in 2018/19) [6]. We previously identified lower uptake by area and amongst some population groups, including minority ethnic groups [7]. Our research in schools with lower uptake showed complex socio-cultural factors influence beliefs and priorities for young women to be vaccinated [8]. The requirement for written parental consent also acts as a major barrier [9], which can be overcome to some extent by enabling verbal parental consent and adolescent self-consent [10][Fisher H, et al, unpublished data].

Based on emerging evidence, the Joint Committee on Vaccination and Immunisation concluded that extending the HPV vaccination programme to include young men could be cost-effective [11]. The Department of Health announced the inclusion of young men from the 2019/20 programme year. If sufficient uptake is attained, a universal vaccination programme will strengthen England's position to eliminate some HPV types and HPV-related cancers in the longer term. Men who have sex with men will also receive optimal protection by immunisation ahead of sexual debut [11, 12].

It is currently unknown whether the universal HPV vaccination programme will be delivered equitably by gender, or whether existing inequalities in uptake and incidence of HPV-related disease among some groups will be decreased or widened [13]. Inequalities may prevent delivery of the programme as intended, with insufficient understanding and engagement of young people and their parents or carers.

For peer review only - http://bmjopen.bmj.com/site/about/guidelines.xhtml

Despite largely positive public attitudes to vaccination [14], recent calls have been made to tackle negative misconceptions of vaccines and limit health misinformation circulating in social media [15]. Many young people have limited or no understanding of the vaccines they receive or the diseases they are intended to prevent [16].

Our previous research confirms some young women attending schools with lower uptake have unmet information needs, which can have implications for obtaining consent and vaccination uptake [8][Fisher H, et al, unpublished data]. A systematic review, including two UK-based studies, showed young men have lower knowledge and understanding of HPV than their female counterparts [17].

Methods to increase understanding, suggested by families and professionals, include: healthcare professional advocacy, accessible information formats, and delivery of educational sessions, in addition to the routine provision of information leaflets [Fisher H, et al, unpublished data]. Preliminary feedback from young people suggests the delivery of interactive HPV vaccine awareness raising sessions by a researcher in the school-setting are acceptable. Young people's questions during the sessions related both to the HPV vaccine and preparation for the vaccination session.

A systematic review suggested weak evidence for the effectiveness of educational interventions at increasing acceptance, or intentions for their daughter to receive the HPV vaccine [18]. However, the review requires updating as the primary studies were predominantly related to hypothetical scenarios prior to vaccine licensing, and more studies have since been published.

Internationally, educational interventions about the HPV vaccine have been developed through research. These have targeted young adults [19, 20], parents [21-23] and professionals [21-23]. A complex intervention delivered in a United States of America healthcare setting, comprising an educational component delivered by healthcare providers to young people aged 11 to 21 years, was shown to be effective at increasing uptake [24]. Similarly, a schools-based educational programme delivered through 113 face-to-face classes with Swedish students aged 16 years was shown to increase self-reported uptake of the HPV vaccine [25]. In Australia, a complex schools-based intervention was

For peer review only - http://bmjopen.bmj.com/site/about/guidelines.xhtml

not shown to be effective at increasing vaccination uptake, but improved adolescent knowledge and psychosocial outcomes [26].

These studies suggest that an educational-based intervention to increase uptake has potential to be an acceptable and effective intervention in the context of the English universal HPV vaccination programme. We hypothesise that mechanisms to address information needs and increase young people's autonomy in consent procedures will result in higher uptake of the programme. We are unaware of any other educational packages that have been developed specifically for areas and populations groups with lower uptake [7], or that have tested these specific mechanisms to increase uptake.

The aim of the study is to co-produce jointly with young people an HPV vaccination educational package that can be used within the adolescent schools-based immunisation programme and is tailored at increasing vaccine uptake in areas and populations with lower HPV vaccination coverage.

METHODS AND ANALYSIS

We propose an innovative, participatory research approach involving co-production of an education package about the HPV vaccine with young people. The 2008 Medical Research Council (MRC) guidance for complex intervention development and evaluation will be followed [27].

Patient and public involvement

The design of this study was informed by our qualitative research undertaken with young women, parents, immunisation nurses and school staff [28][Fisher H, et al, unpublished data]. We have consulted with the Bristol Young People's Advisory Group (YPAG) and Bristol City Youth Council about the design of the study. The Bristol YPAG comprises young people aged ten to 17 years who are interested in healthcare and research. The Bristol City Youth Council are an elected group of 28 young people from across the city. Both groups have been consulted about the design of the study and participant materials. They will also be invited to an event at the end of the study to consider findings and recommendations with the young people, healthcare professionals and school staff involved in the study.

For peer review only - http://bmjopen.bmj.com/site/about/guidelines.xhtml

The study will comprise three phases: (1) a rapid review of adolescent immunisation information materials and preliminary qualitative interviews with young people and key stakeholders; (2) theory development and co-production of HPV vaccine communication materials in an iterative process with young people, and; (3) examining delivery mechanisms and acceptability of the educational package in four schools with lower uptake of the HPV vaccination programme.

Recruitment

During Stages One and Two, young men and women aged 12 to 15 years will be recruited from two youth community organisations in the south west of England. These organisations work with young people with similar sociodemographic backgrounds to the participants of our previous studies undertaken in schools with lower uptake. An additional youth community organisation in London has also been recruited. The age group 12 to 15 years is selected as these young people will have diverse experiences in relation to the HPV vaccination programme. Key stakeholders will initially be identified through the research teams existing relationships with youth organisations, immunisation teams and schools in the study areas. Additional interviews may be undertaken if other key individuals are identified as the activities progress,

Stage One

A scoping review of existing young people's immunisation information materials will be undertaken. Web-based searches will be undertaken to locate online HPV vaccine communication tools targeted at young people in English speaking countries. A description of the characteristics, format, and content of the communication tools will be provided. The findings will be used to make recommendations to inform the content of the initial workshop.

Our qualitative research findings suggest that young people value external providers delivering health-related information in school settings. Preliminary individual or paired/peer group interviews (depending on the preference of participants) with young people (n~6) and key stakeholders (commissioners, school staff, immunisation nurses and youth workers/practitioners) (n~6) will be undertaken to explore further who should deliver the educational package and which key messages to cover.

For peer review only - http://bmjopen.bmj.com/site/about/guidelines.xhtml

Stage Two

Drawing on our previous qualitative research findings, it is envisaged that the educational package will include question and answer sessions alongside a series of short videos. Information needs may differ between schools depending on the sociodemographic characteristics of the student population. Therefore, additional videos will be co-produced to allow the educational session to be tailored as appropriate (e.g. provision of information in multiple languages, additional information for parents on vaccine safety).

The materials will be co-produced by young people (n~6) and creative designers who are experienced in working with young people to create health-related media materials.

An initial workshop with young people (n~6-8) will be organised at a community youth organisation where they will be asked to: (i) review existing communication materials; (ii) comment on their understanding of key HPV vaccine messages; (iii) make suggestions of their preferred messages, design and language style, and; (iv) propose the type of media to be used (e.g. animation, vloggers). Platforms to distribute the communication materials (e.g. videos, power-points) which are accessible by young people will be explored. Advice will also be sought from voluntary organisations, immunisation teams and school staff.

At least three additional workshops will be organised in different community youth organisations. Here the materials will be iteratively redesigned by the co-production team. Young people's views will be taken into account at all stages of the development cycle.

Stage Three

Four schools with lower uptake of the HPV vaccination programme will be recruited to the study. Identification of potential schools to approach will be made in collaboration with the local immunisation teams in Bristol and London and our existing relationships with schools. It is envisaged that the educational package will last approximately one hour and be delivered to groups of vaccine eligible young people (aged 12-13 years). The package will be piloted approximately two weeks ahead of the

For peer review only - http://bmjopen.bmj.com/site/about/guidelines.xhtml

scheduled vaccination session. Methods for obtaining young people's feedback will be explored (e.g. short paper-based questionnaires, interactive quiz). Following the question and answer session, young people will be provided with consent forms and promotional materials available in different languages. Young people will be encouraged to share these with their parents/carers and return the completed consent form.

The feasibility of obtaining information from immunisation teams relating to HPV vaccine uptake by gender, ethnicity, deprivation, and types of consent routinely obtained (no response, parent refusal, written or verbal parental consent, young person refusal or self-consent) will be assessed in terms of timeliness of data acquisition and levels of completeness for a future study.

A purposive sample of young people (~12) from participating schools and key stakeholders (n~12) will be invited to take part in semi-structured or paired/peer group interviews (depending on the preference of participants) to gather information related to intervention acceptability, understanding of the HPV vaccine, and perceptions of adolescent autonomy during consent. Potential key stakeholders to invite to interview will be identified by the research team as the research activities progress. The feasibility and sustainability of who delivers the education package will be further explored.

Analysis

All interview recordings will be transcribed verbatim and any potentially identifying information removed. Familiarisation with the data will involve two researchers reading and discussing the transcripts to compare and begin to code and categorise the data. Thematic analysis [29] will be undertaken, within which similarities and differences will be explored. Data analysis will be assisted by the QSR NVivo12 software package.

Together, the findings from the rapid review and qualitative research will inform the logic model and development of theory for the educational package.

The outcome from this participatory research study will be a tailored educational intervention about the universal HPV vaccination programme. We will seek additional funding to test whether the educational

For peer review only - http://bmjopen.bmj.com/site/about/guidelines.xhtml

package can: (i) improve uptake of the vaccination programme in schools/areas with lower uptake; (ii) reduce inequalities in programme delivery; (iii) increase young people's understanding about the HPV vaccine, and; (iv) increase young people's autonomy in decision-making and consent procedures. For this larger scale study, we plan to design an intervention based on behaviour change theory (e.g. COM-B model) which uses delivery of the educational materials we will have co-produced alongside other strategies to support behaviour change.

Ethics and dissemination

In March 2020, the University of Bristol's Faculty of Health Sciences Research Ethics Committee have provided ethical approvals for the study (reference: 99102). The project began recruitment and data collection in April 2020 and the studies activities will finish in March 2021.

We will disseminate this research to the academic community, health and youth work practitioners and health policy workers. For the academic community we plan to submit at least one peer-reviewed paper. We will also disseminate the results to at least one national and one international conference. For practitioners we will complete a report of our findings and will disseminate the results through practitioner conferences and events.

We will also hold a community stakeholder event to which we will invite school nurses, school staff, parents and young people. This will be an opportunity to discuss the educational package for the HPV vaccine. Healthcare professionals will be actively encouraged to be involved in the dissemination activities, and findings from the study will also be disseminated through appropriate websites and social media. The final report of the study will also provide an opportunity for further dissemination.

REFERENCES

1. Drolet, M., et al., Population-level impact and herd effects following the introduction of human papillomavirus vaccination programmes: updated systematic review and meta-analysis. The Lancet, 2019. **394**(10197): p. 497-509.

2. Simms, K., et al., Impact of scaled up human papillomavirus vaccination and cervical screening and the potential for global elimination of cervical cancer in 181 countries, 2020-99: a modelling study. The Lancet Oncology, 2019. **20**(3): p. 394-407.

3. Johnson, H., et al., Effect of HPV vaccination and cervical cancer screening in England by ethnicity: a modelling study. The Lancet Public Health, 2018. **3**(1): p. e44-e51.

For peer review only - http://bmjopen.bmj.com/site/about/guidelines.xhtml

4. Baker, D. and E. Middleton, Cervical screening and health inequality in England in the 1990s. 2003. **57**(6): p. 417-423.

5. Shack, L., et al., Variation in incidence of breast, lung and cervical cancer and malignant melanoma of skin by socioeconomic group in England. BMC Cancer, 2008. **8**(1): p. 271.

6. Public Health England, HPV vaccination coverage in adolescent females in England: 2018 to 2019, 2019. Avaialble from: https://www.gov.uk/government/statistics/hpv-vaccination-coverage-in-adolescent-females-in-england-2018-to-2019.

7. Fisher, H., et al., Examining inequalities in the uptake of the school-based HPV vaccination programme in England: a retrospective cohort study. Journal of Public Health, 2013. **36**(1): p. 36-45.

8. Batista Ferrer, H., et al., Barriers and facilitators to uptake of the school-based HPV vaccination programme in an ethnically diverse group of young women. Journal of Public Health, 2016. **38**(3): p. 569-577.

9. Batista Ferrer, H., Factors influencing the uptake of the Human Papillomavirus (HPV) vaccination programme. Thesis (Ph.D.), 2014.

10. Audrey, S., et al., How acceptable is adolescent self-consent for the HPV vaccination: qualitative findings from a process evaluation in south-west England. Vaccine, 2020. https://doi.org/10.1016/j.vaccine.2020.09.074

11. Joint Committee on Vaccination and Immunisation, 2018. Available from: https://www.gov.uk/government/publications/jcvi-statement-extending-the-hpv-vaccination-programme-conclusions.

12. Kesten, J., Flannagan, C, Ruane-McAteer, E, Merriel, S, Nadarzynski, T, Shapiro, G, Rosberger, Z & Prue, G, A mixed-methods study in England and Northern Ireland to understand young men-who-have-sex-with-men's knowledge and attitude towards human papillomavirus vaccination. BMJ Open, 2019. **9**: p. e025070.

13. Department of Health & Social Care, Equality Analysis: Human Papillomavirus (HPV) Vaccination, 2018. Available from: https://assets.publishing.service.gov.uk/government/uploads/system/uploads/attachment_data/file/758777/HPV_Boys_Equality_Assessment.pdf.

14. Campbell, H., et al., Changing attitudes to childhood immunisation in English parents. Vaccine, 2017. **35**(22): p. 2979-2985.

15. Royal Society for Public Health, Moving the needle: promoting vaccination uptake across the life course, 2018. Available from - https://www.rsph.org.uk/uploads/assets/uploaded/f8cf580a-57b5-41f4-8e21de333af20f32.pdf.

16. Davies, C., et al., 'Is it like one of those infectious kind of things?' The importance of educating young people about HPV and HPV vaccination at school. Sex Education, 2017. **17**(3): p. 256-275.

17. Prue, G., et al., Knowledge and acceptance of human papillomavirus (HPV) and HPV vaccination in adolescent boys worldwide: A systematic review. Journal of Cancer Policy, 2016. **10**: p. 1-15.

18. Fu, L., et al., Educational interventions to increase HPV vaccination acceptance: a systematic review. Vaccine, 2014. **32**(17): p. 1901-1920.

19. Ayoub, N., J. Sunwoo, and H. Starmer, Implementation of a targeted HPV educational program in a population with HIV. World Journal of Otorhinolaryngology - Head and Neck Surgery, 2019.

For peer review only - http://bmjopen.bmj.com/site/about/guidelines.xhtml

20. Chang, I., et al., Effect of an educational intervention on HPV knowledge and vaccine attitudes among urban employed women and female undergraduate students in China: a cross-sectional study. BMC Public Health, 2013. **13**(1): p. 916.

21. Reiter, P., et al., HPV and HPV vaccine education intervention: effects on parents, healthcare staff, and school staff. Cancer epidemiology, biomarkers & prevention : a publication of the American Association for Cancer Research, cosponsored by the American Society of Preventive Oncology, 2011. **20**(11): p. 2354-2361.

22. Dempsey, A., et al., Effect of a Health Care Professional Communication Training Intervention on Adolescent Human Papillomavirus Vaccination: A Cluster Randomized Clinical Trial. JAMA Pediatrics, 2018. **172**(5): p. e180016-e180016.

23. Paskett, E., et al., Results of a Multilevel Intervention Trial to Increase Human Papillomavirus (HPV) Vaccine Uptake among Adolescent Girls. Cancer Epidemiology Biomarkers & Prevention, 2016. **25**(4): p. 593-602.

24. Perkins, R., et al., Effectiveness of a provider-focused intervention to improve HPV vaccination rates in boys and girls. Vaccine, 2015. **33**(9): p. 1223-9.

25. Grandahl, M., et al., School-based intervention for the prevention of HPV among adolescents: a cluster randomised controlled study. BMJ Open, 2016. **6**(1): p. e009875.

26. Skinner, S., et al., LB1.1 Randomised controlled trial of a complex intervention to improve school-based hpv vaccination for adolescents: the hpv. edu study. Sexually Transmitted Infections, 2015. **91**(Suppl 2): p. A77-A77.

27. Craig, P., et al., Developing and evaluating complex interventions: the new Medical Research Council guidance. BMJ (Clinical research ed.), 2008. **337**: p. a1655-a1655.

28. Audrey, S., et al., Impact and acceptability of self-consent procedures for the school-based human papillomavirus vaccine: a mixed-methods study protocol. BMJ Open, 2018. **8**(3): p. e021321.

29. Braun, V. and V. Clarke, Using thematic analysis in psychology. Qualitative Research in Psychology, 2006. **3**(2): p. 77-101.

For peer review only

For peer review only - http://bmjopen.bmj.com/site/about/guidelines.xhtml

Data statement

It is the authors' intention to share their underpinning research data in order to maximise reuse and evidence their findings. When participants consent or assent to take part in the study, their permission to deposit anonymised transcripts at the University of Bristol Research Data Repository (data.bris.ac.uk/data) will be sought. A metadata record will be published openly by the repository and this record will clearly state how data can be accessed by bona fide researchers. Requests for access will be directed to the Research Data team at Bristol, who will assess the motives of potential data re-users before granting access to the data. No authentic request for access will be refused and re-users will not be charged for any part of this process.

Author statement

All authors were involved in the conception and design of the research: MH is Principal Investigator and contributes expertise in researching infectious disease control; SA contributes expertise in qualitative research and process evaluation. TC provides implementation, mixed methods and anthropology research expertise in relation to UK vaccination programmes. AF provides experience in the field of vaccine and policy development. LL provides expertise in implementing the national HPV vaccination programme. SM-J has expertise in health systems and policy evaluations, notably vaccination programmes. CT offers expertise in knowledge mobilisation. JY will support the dissemination of findings and wider implementation of the educational package as appropriate. As study manager, HF will be responsible for co-ordinating the study and provides links with community organisations and schools. HF wrote the first draft and all authors contributed to the final version of the manuscript.

Funding

This work is supported by the Medical Research Council (MRC) Public Health Intervention Development scheme (project number: MR/T027150/1).

Acknowledgments

The study is supported by the NIHR Health Protection Research Unit in Behavioural Science and Evaluation at the University of Bristol. The views expressed are those of the authors and not necessarily those of the NHS, the NIHR, the Department of Health or Public Health England.

For peer review only - http://bmjopen.bmj.com/site/about/guidelines.xhtml

This study is supported by the National Institute for Health Research (NIHR) Health Protection Research Unit in Vaccines and Immunisation, a partnership between Public Health England and the London School of Hygiene and Tropical Medicine. The views expressed are those of the author(s) and not necessarily those of the NIHR, Public Health England or the Department of Health and Social Care.

Conflicts of interest

The authors declare there are no conflicts of interest.

For peer review only

For peer review only - http://bmjopen.bmj.com/site/about/guidelines.xhtml
